# Effective Deconstruction of Lignocellulose Through Oxidative Catalytic Fractionation Under Additive-Free Non-Alkaline System via Co-LDO Catalyst

**DOI:** 10.3390/polym18080922

**Published:** 2026-04-09

**Authors:** Haozhi Zhang, Wei Yan, Ying Wang, Cheng-Ye Ma, Changfu Zhuang

**Affiliations:** 1 International Joint Research Center for Biomass Materials, Southwest Forestry University, Kunming 650051, China; 2 Department of Biosystems Engineering, Zhejiang University, 866 Yuhangtang Road, Hangzhou 310058, China; 3 Institute of Zhejiang University-Quzhou, 99 Zheda Road, Quzhou 324000, China

**Keywords:** oxidative catalytic fractionation, lignin, carbohydrates, Co-LDO, poplar wood

## Abstract

Oxidative catalytic fractionation (OCF) under the lignin-first strategy has emerged as a critical technological approach for biomass refining. To address the inevitable carbohydrate degradation and lignin condensation in conventional OCF, this study designed a cobalt-doped layered double hydroxide oxide (Co-LDO) catalyst compatible with non-alkaline (without Brønsted bases) organic systems, which exhibits excellent performance in poplar biomass OCF. With a straightforward preparation process, the Co-LDO catalyst yields high-content oxidized lignin oligomers while efficiently retaining carbohydrates, providing feedstock rich in carbohydrates (cellulose and hemicellulose) for the subsequent production of bioenergy and biomass-based chemicals. Under optimized conditions screened via systematic reaction condition investigation and metal-doped LDO catalyst evaluation, the process achieved a 94.01 wt% delignification rate, with 72.19 wt% of lignin converted into lignin oligomer oil, supported by detailed product composition and structural characterization. Meanwhile, 74.14 wt% hemicellulose and 98.23 wt% cellulose were recovered in solid residues, with structurally intact hemicellulose retention being 2.3 times higher than in traditional OCF. Mass balance calculation confirmed a total poplar refining yield of 81.58 wt%. In summary, this Co-LDO-catalyzed OCF strategy provides a high-activity non-precious metal system, effectively suppressing lignin condensation while preserving high-yield carbohydrates, realizing the efficient full-component refining of poplar biomass.

## 1. Introduction

Faced with the challenges of global warming and the rapid deterioration of the ecological environment, replacing increasingly depleted fossil resources with renewable energy to fulfill societal energy demands and improve the global ecological environment has become a significant task [1,2,3]. Lignocellulose biomass constitutes a vital renewable resource, primarily composed of three major components: lignin, cellulose, and hemicellulose [4,5,6]. It serves as a crucial upstream feedstock for the production of environmentally friendly materials, chemicals, and energy fuels [7,8,9,10,11,12]. Biomass conversion technology possesses carbon-neutral attributes, aligning closely with the current societal drive towards green production and low-carbon energy [6,13,14]. Traditional lignocellulose-refining technology focuses on the efficient separation of components, with particular emphasis on the enrichment and recovery of high-value carbohydrates [5,15,16]. Macromolecular carbohydrates have many uses; for example, cellulose has long been used to produce pulp, while hemicellulose can be converted into valuable products such as xylitol and polysaccharides [9,17,18]. However, the lignin generated during this process is difficult to convert into high-value products, with most being discarded or incinerated directly, thereby failing to achieve efficient energy recovery [17,19,20,21]. Against this backdrop, the lignin-first strategy has emerged [13,22,23]. This strategy achieves the dissociation of biomass while simultaneously enabling the directed catalytic degradation of lignin into oligomers and even phenolic monomers, while effectively inhibiting carbohydrate degradation [2,24,25]. The carbohydrate-rich solids (containing cellulose and hemicellulose) obtained after the lignin removal process can be further processed and recycled as macromolecular polymers. This prevents the waste of resources that occurs when carbohydrates are broken down into small-molecule and low-value substances during the implementation of the lignin-first strategy. This highly efficient conversion technology can significantly reduce lignocellulose waste and substantially enhance the comprehensive utilization efficiency of biomass [7,26,27]. The coupled strategy of biomass deconstruction and catalytic fractionation conversion not only improves the energy utilization efficiency but also increases products’ added value, thereby attracting widespread attention from researchers in this field [2,28].

**Figure 1:**
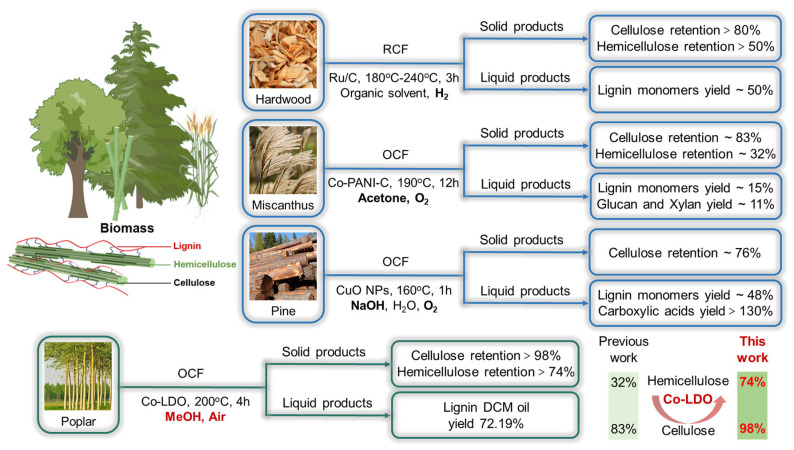
Schematic diagrams of traditional lignin-first strategy and catalytic oxidation fractionation (Co-LDO).

The lignin-first strategy is mainly divided into two technological pathways [7]: reductive catalytic fractionation (RCF) and oxidative catalytic fractionation (OCF) [6,22,24,29,30]. Its core aim is to dissociate lignin from biomass efficiently and convert it into soluble fractions catalytically, such as phenolic monomers and oligomers, while retaining part of the mixed cellulose as an insoluble fraction [5,24]. Although this strategy has been developed over many years, numerous technical bottlenecks remain to be urgently overcome [8]. Specifically, RCF suffers from drawbacks including a reliance on precious metal catalysts and excessive hydrogen consumption [2,13]. Conversely, while OCF eliminates hydrogen and precious metal use, it is affected by excessive carbohydrate degradation [31,32]. As demonstrated in Figure 1, prior studies have shown that traditional hardwoods react with catalysts such as Ru/C and Pd/C within heterogeneous catalytic systems (organic solvents and H_2_) [2,6,33]. Lignin is subjected to in situ dissociation and catalytic conversion to produce phenolic monomers, with a yield of 50 wt% based on the original lignin content in the biomass. In this process, the retention rate of cellulose in carbohydrates exceeds 80%, while that of hemicellulose is only 50%. Both figures are calculated based on the mass of carbohydrates present in the biomass [24]. To avoid the use of hydrogen and precious metal catalysts, the emerging oxidative catalytic fractionation (OCF) technology has advanced rapidly in recent years [23,34,35]. Stahl’s group designed a heterogeneous non-precious metal Co-N-C catalyst for the oxidative catalytic fractionation of Miscanthus using acetone as a solvent in an oxygen atmosphere [28]. The soluble fraction obtained after 12 h of reaction at 190 °C contained 15 wt% lignin phenolic monomers and 11 wt% glucan and xylose, while the solid residue retained only 82 wt% cellulose and 32 wt% hemicellulose. Liao’s group employed CuO NPs as a catalyst for the oxidative separation of pine biomass in an aqueous sodium hydroxide–oxygen (NaOH-O_2_) system [18]. After treating pine at 160 °C for 1 h, the soluble fraction yielded 48 wt% lignin phenolic monomers and 130 wt% carboxylic acids. Concurrently, cellulose retention exceeded 80 wt%, and pulp purity reached 95%. Multiple studies have confirmed that the lignin-first catalytic fractionation strategy faces a large number of challenges in recovering carbohydrates. The cellulose yields from the RCF and OCF processes are comparable, and some cellulose is degraded in both types of processes [18,28,36]. However, among the various OCF methods using either alkaline water or organic solvents, excessive hemicellulose degradation occurs [18]. Moreover, hemicellulose is largely difficult to recover effectively as a solid component, resulting in the significant waste of natural hemicellulose resources [18].

Therefore, to address the aforementioned challenges and improve the overall efficiency of biomass oxidative catalysis, this study developed a cobalt-doped double-layer oxide (Co-LDO) catalyst under an additive-free non-alkaline system for the lignin-first OCF strategy using poplar as the feedstock. This strategy aimed to achieve the efficient component dissociation of poplar biomass and the catalytic conversion of lignin, while protecting cellulose and hemicellulose from degradation during the OCF process. Various non-precious metal-doped LDO catalysts were synthesized using a simple coprecipitation method. Their physicochemical properties were systematically characterized and evaluated using characterization techniques including X-ray diffraction (XRD), X-ray photoelectron spectroscopy (XPS), physical adsorption, CO_2_ temperature-programmed desorption (CO_2_-TPD), and transmission electron microscopy (TEM). Using the lignin dichloromethane oil (lignin DCM oil) yield as the criterion, different reaction conditions (temperature, time, gas pressure, catalyst dosage, solvent, and molar ratio) were screened. Subsequently, different metal-doped LDO catalysts were prepared to investigate the catalytic conversion efficiency and patterns of poplar under optimal reaction conditions. Advanced characterization techniques (including HPLC, 2D-HSQC, GC-MS, and GPC, etc.) were employed to systematically evaluate the resulting lignin DCM oil and the extracted hemicellulose. In summary, this study proposes a more cost-effective and efficient Co-LDO catalytic system that efficiently catalyzes the degradation of poplar components while selectively retaining carbohydrates. This provides new theoretical and technical support for the optimization of lignin-first refining strategies.

## 2. Materials and Methods

### 2.1. Materials

Poplar sourced from fast-growing poplar trees in Hebei Province was ground into a powder and sieved to produce 40–60-mesh particles. Methanol, ethanol, ethylene glycol, glycerol, tetrahydrofuran, n-decane, dichloromethane, Na_2_SO_4_, Na_2_CO_3_, NaOH, Co(NO_3_)_2_·2.5H_2_O, Cu(NO_3_)_2_·3H_2_O, Fe_2_(NO_3_)_3_·9H_2_O, Ni(NO_3_)_2_·6H_2_O, AlCl_3_·6H_2_O, MgCl_2_·6H_2_O, dimethylsulfoxide-*d*_6_ (DMSO-*d*_6_), NaOD, and other chemical reagents were purchased from Aladdin Biochemical Technology Co., Ltd. (Shanghai, China). All reagents were used directly, without further purification.

### 2.2. Synthesis of Co-LDO

The catalyst prepared in this procedure was a porous metal oxide, denoted as Co-LDO, which indicates that, in a 3:1 Mg/Al layered double hydroxide (LDH) precursor, some of the Mg^2+^ ions were replaced with Co^2+^ ions, and Co-LDO was prepared by coprecipitation. An LDH precursor for the preparation of 20% Co^2+^ ions instead of Mg^2+^ ions was introduced as Co-LDH. In a typical procedure, a solution containing AlCl_3_·6H_2_O (7.25 g, 0.15 mol), Co(NO_3_)_2_·2.5H_2_O (5.24 g, 0.09 mol), and MgCl_2_·6H_2_O (14.64 g, 0.36 mol) in deionized water (200 mL) was added to a solution containing Na_2_CO_3_ (12.72 g, 0.12 mol) in water (200 mL) at 60 °C under 700 rpm stirring with a speed of two drops per second. The pH was kept between 9.5 and 10.5 by the addition of small portions of a 2 mol/L solution of NaOH, and it was stirred at 60 °C for 12 h. After cooling to room temperature, the pink solids were filtered and washed with deionized water and ethanol, followed by drying the solid for 12 h at 80 °C. Before use, Co-LDH was calcined at 500 °C for 6 h in air with a temperature ramp of 5 °C/min to obtain the Co-LDO catalyst. All other metal LDOs (MgAl-LDO, Cu-LDO, Ni-LDO, Fe-LDO, Co-LDO) were prepared by this method, and the Co molar ratio described in the following refers to the ratio of substituted Mg^2+^ ions. All experiments were performed in triplicate, and the data were expressed as the mean standard deviation.

### 2.3. Catalyst Characterization

X-ray diffraction (XRD) data were collected using the Bruker D8 Advance diffractometer (Bruker Corporation, Billerica, MA, USA) (Cu Kα, 5–90°, 5° min^−1^). X-ray photoelectron spectroscopy (XPS) experiments were conducted with the Thermo Escalab 250Xi spectrometer (Thermo Fisher Scientific, Waltham, MA, USA) (Al kα, hv = 1486.6 eV). The BJH pore volumes and BET-specific surface areas of samples were determined using the Kubo X1000 apparatus (Builder Electronic Technology Co., Ltd., Beijing, China). Temperature-programmed desorption of CO_2_ (CO_2_-TPD) experiments were performed on the Altamira Instruments AMI-300 apparatus (Altamira Instruments, LLC, Cumming, GA, USA). Transmission electron microscopy (TEM) was performed on the JEOL JEM-F200 apparatus (JEOL Ltd., Tokyo, Japan) (200 kV, JED-2300T).

### 2.4. Oxidative Catalytic Fractionation of Poplar

Typically, the autoclave was charged with 0.1 g Co-LDO catalyst, 1 g poplar, and 20 mL methanol and pressurized with 1.5 MPa air at room temperature. Then, the reactor (Shanghai LABE Instrument Co., Ltd., Shanghai, China) was heated to 200 °C for a predetermined amount of time and stirred at 700 rpm, and it was subsequently cooled to room temperature. In the reactor, the mixture was collected for solid–liquid separation and washed three times with methanol; the liquid was spun dry under a vacuum and extracted with dichloromethane and deionized water. The organic phase was vacuum spin-dried and weighed to obtain the lignin dichloromethane extract oil, named lignin DCM oil. The separated solids were dried to a constant weight to achieve solid recovery. Subsequently, the solid mixture was sieved through a 60-mesh sieve, followed by further extraction with ethyl acetate and water to achieve the thorough separation of the catalyst from the carbohydrate-rich biomass. The poplar solid was subjected to NREL analysis to assess the cellulose, hemicellulose, and lignin quality and then calculate solid recovery, delignification, cellulose retention, and hemicellulose retention; the NREL method is described in the Supplementary Materials, Section S1.5. The control group without an added catalyst was named Con, and the group with different LDO catalysts added was named Metal-LDO. In this experiment, the reaction was scaled up 40 times and conducted in a 2 L reactor. Samples were taken after the OCF reaction to calculate the yields of each product, resulting in the mass balance.

The cellulose retention, hemicellulose retention, and lignin recovery were calculated as follows:

$$\mathrm{Lignin}\;\mathrm{DCM}\;\mathrm{oil}\;(\mathrm{wt}\%)\;=\;\frac{\mathrm{DCM}\;\mathrm{extracted}\;\mathrm{oil}\;\mathrm{quality}}{\mathrm{poplar}\;\mathrm{lignin}\;\mathrm{quality}}$$

$$\mathrm{Solid}\;\mathrm{recovery}\;(\mathrm{wt}\%)=\frac{\mathrm{OCF}-\mathrm{treated}\;\mathrm{solid}\;\mathrm{quality}-\mathrm{catalyst}\;\mathrm{quality}}{\mathrm{poplar}\;\mathrm{quality}}$$

$$\mathrm{Delignification}\;(\mathrm{wt}\%)=1-\frac{\mathrm{lignin}\;\mathrm{quality}\;\mathrm{after}\;\mathrm{OCF}\;\times \;\mathrm{solid}\;\mathrm{recovery}}{\mathrm{poplar}\;\mathrm{lignin}\;\mathrm{quality}}$$

$$\mathrm{Cellulose}\;\mathrm{retention}\;(\mathrm{wt}\%)=\frac{\mathrm{cellulose}\;\mathrm{quality}\;\mathrm{after}\;\mathrm{OCF}}{\mathrm{poplar}\;\mathrm{cellulose}\;\mathrm{quality}\;\times \;\mathrm{solid}\;\mathrm{recovery}}$$

$$\mathrm{Hemicellulose}\;\mathrm{retention}\;(\mathrm{wt}\%)=\frac{\mathrm{hemicellulose}\;\mathrm{quality}\;\mathrm{after}\;\mathrm{OCF}}{\mathrm{poplar}\;\mathrm{hemicellulose}\;\mathrm{quality}\;\times \;\mathrm{solid}\;\mathrm{recovery}}$$

$$\mathrm{Monomer}\;\mathrm{yield}\;(\mathrm{wt}\%)=\frac{\mathrm{monomer}\;\mathrm{quality}}{\;\mathrm{poplar}\;\mathrm{lignin}\;\mathrm{quality}\;}$$

### 2.5. Lignin Fraction Analysis

Gel permeation chromatography (GPC, Agilent 1200, Agilent Technologies, Inc., Santa Clara, CA, USA) was employed to characterize the molecular weight (M_w_) of lignin DCM oil. A Bruker AVIII 400 MHz spectrometer (Bruker Corporation, Ettlingen, Germany) was utilized with a deuterated solvent (DMSO-*d*_6_) to obtain the 2D-HSQC spectrum of lignin, observing the functional groups in its aromatic and side-chain regions. To obtain quantitative data for lignin monomers, n-decane was added as an internal standard to the lignin DCM oil. Following dissolution in methanol and filtration through a 0.22 μm filter, the sample was subjected to qualitative analysis via GC-MS (Agilent 8860 GC system equipped with an HP-5 MS column and Agilent 5977C mass spectrometer detector, Agilent Technologies, Inc., Santa Clara, CA, USA). Furthermore, quantitative analysis was performed using GC-FID (Agilent 8860 GC system equipped with an HP-5 column and a flame ionization detector (FID), Agilent Technologies, Inc., Santa Clara, CA, USA).

### 2.6. Characterization of Hemicellulose Component

Hemicellulose was extracted from the solid residue after OCF by the alkaline extraction–ethanol precipitation method: 1 g solid residue was added to 50 mL 10 wt% KOH solution and reacted at 50 °C for 3 h under a nitrogen atmosphere. The mixture was filtered, and the filtrate was neutralized with glacial acetic acid to pH 5.5 and then concentrated by rotary evaporation, and 3 times the volume of anhydrous ethanol was added to precipitate hemicellulose. The precipitate was washed with 70 wt% ethanol 3 times and freeze-dried to obtain a purified hemicellulose sample. Gel permeation chromatography was employed to characterize the average molecular weight (M_n_) and number-average molecular weight (M_w_) of hemicellulose. The molecular structure of hemicellulose was characterized by 2D-HSQC NMR on a Bruker AVIII 400 MHz spectrometer. An approximately 50 mg hemicellulose sample was dissolved in 0.5 mL D_2_O with a small amount of NaOD to enhance the solubility. The number of scans was 32, the relaxation time was 1.5 s, and the spectral widths were 5000 Hz for the ^1^H dimension and 20,000 Hz for the ^13^C dimension.

## 3. Results and Discussion

### 3.1. Characterization of Co-LDO Catalyst

**Figure 2:**
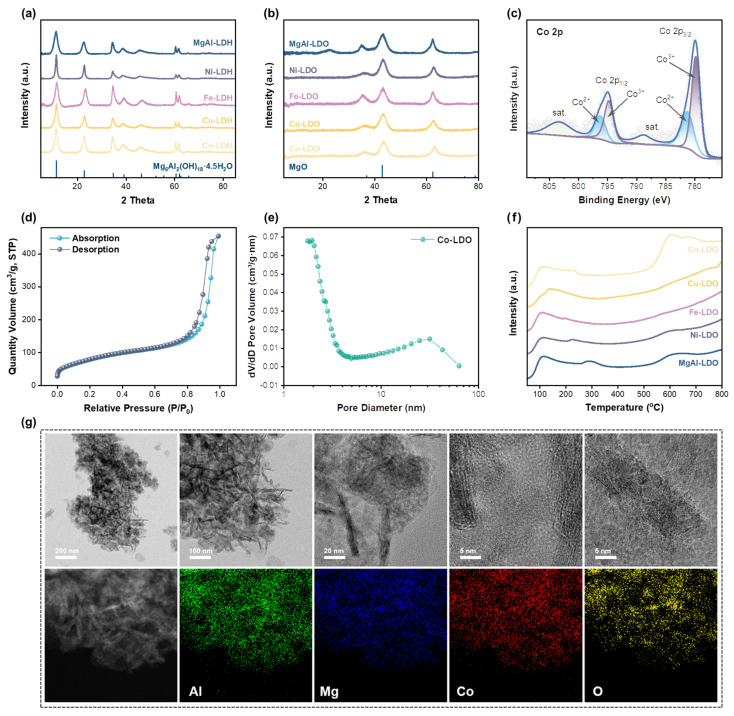
(**a**) XRD patterns of all LDH catalysts; (**b**) XRD patterns of all LDO catalysts; (**c**) XPS spectra of Co 2p; (**d**) BET adsorption–desorption isotherm and (**e**) pore size distribution of Co-LDO catalyst; (**f**) CO_2_-TPD profiles for all LDO catalysts; (**g**) TEM image and EDS element-mapping analysis of Al, Mg, Co, O distribution.

The LDO catalysts were synthesized via the calcination of LDH precursors, following the previously reported procedure. All LDH precursors and LDO catalysts were characterized by XRD [24]. The results were in excellent agreement with the theoretical predictions, confirming that the metal ions were fully incorporated into the host hydrotalcite lattice. Powder XRD measurements verified the formation of the LDH structure during precursor synthesis (Figure 2a). Consistent with the literature data [24], these samples exhibited sharp diffraction peaks at 11.4°, 23.21°, and 34.73°, accompanied by broad diffraction peaks at 39.20°, 46.50°, 52.44°, 60.61°, and 61.81°, which were consistent with the characteristic diffraction peaks of the trigonal hydrotalcite phase [Mg_6_Al_2_(OH)_18_·4.5H_2_O, JCPDS Card No. 35-0965]. Subsequently, the synthesized LDHs were calcined at 500 °C for 6 h, resulting in the transformation of the layered hydrotalcite structure into an amorphous mixed oxide phase. Three prominent diffraction peaks centered at 37.0°, 42.0°, and 62.3° were indexed to magnesia (MgO, JCPDS Card No. 45-0946). Notably, no diffraction peaks attributable to Co_2_O_3_ or other metal oxides were detected, as shown in Figure 2b, confirming the homogeneous dispersion of the active metal species. All sharp diffraction peaks exhibited varying degrees of shift, presumably due to the incorporation of active metals into the alumina matrix. Furthermore, the diffraction profiles of other LDO catalysts differed from those of LDH and MgAl-LDO, stemming from the substitution of Mg^2+^ ions in the mixed oxide lattice by other active metal ions.

XPS measurements were carried out on the Co-LDO catalyst to gain deeper insight into the chemical state of the active species. As shown in Figure 2c, two main peaks at 779.9 eV and 795.0 eV, corresponding to Co 2p_3_/_2_ and Co 2p_1_/_2_, respectively, appeared in the Co high-resolution XPS spectrum, which were typical spin–orbit splitting peaks of Co_3_O_4_. The binding energy difference (ΔE = E(Co 2p_1_/_2_) − E(Co 22p_3_/_2_)) between the two peaks was 15.1 eV, with a peak intensity ratio of approximately 2:1. The peaks at 779.8 eV and 794.8 eV corresponded to Co^3+^ species, while the peaks at 781.3 eV and 796.3 eV were assigned to typical Co^2+^ species. Additionally, the satellite peaks observed at 788.9 eV and 803.5 eV further confirmed that cobalt oxide existed in the form of Co_3_O_4_ [37]. The above results verify that the active cobalt species doped in the Co-LDO catalyst existed in the form of Co_3_O_4_, which provided the redox active sites for lignin oxidative degradation.

The porous structures of the LDO catalysts were systematically investigated via nitrogen adsorption–desorption (BET) measurements [38]. Figure 2d,e depict the adsorption–desorption isotherm and pore size distribution curve of Co-LDO, respectively. The results revealed a typical reversible type IV isotherm with an H4-type hysteresis loop, a signature characteristic of mesoporous materials. The pore size distribution curve exhibits two distinct characteristic peaks at 1.95 nm (microporous range, <2 nm) and 31.64 nm (large mesoporous range), corresponding to microporous and large mesoporous structures, respectively. Further characterization demonstrated that Co-LDO possessed a large BET-specific surface area (285.77 m^2^·g^−1^), a low pore volume (0.11 cm^3^·g^−1^), and a small average pore size (9.46 nm). This structural feature was presumably attributed to the Co doping-induced lattice reconstruction of Mg_3_AlO_x_, which facilitated the conversion of the original large mesopores into smaller mesoporous structures. Additionally, the LDO catalysts with different Co molar doping ratios were all mesoporous materials, exhibiting distinct BET-specific surface areas owing to variations in the Co doping concentration. The large specific surface area and hierarchical porous structure can provide abundant exposed active sites, facilitate contact between lignin macromolecules and active sites, and reduce mass transfer resistance during the reaction, which are beneficial to the catalytic degradation of lignin.

The basic sites of LDO catalysts were characterized by CO_2_-TPD, as they are a key factor affecting the OCF performance of lignocellulose [37]. The CO_2_-TPD profiles and total desorbed CO_2_ amounts of the samples are presented in Figure 2f. The variation in the CO_2_ desorption peaks with temperature revealed that the LDO catalysts mainly possessed three types of typical basic sites: weak basic sites (50–250 °C), medium basic sites (250–500 °C), and strong basic sites (>500 °C). Tests on catalyst samples of the same mass demonstrated that all LDO catalysts exhibited broad CO_2_ desorption peaks in the range of 50–250 °C, with sharp peak shapes around 100 °C. This weak basic peak originated from the intrinsic basic sites provided by the MgO active component in the pristine MgAl-LDO support. Furthermore, detailed analysis showed that, as the temperature increased, the CO_2_ desorption capacity of the LDO catalysts decreased. Only MgAl-LDO displayed a relatively weak desorption peak in the medium basic site range (250–500 °C), whereas, for other metal-doped LDO catalysts, the partial substitution of Mg by doped metals led to a reduction in the number and migration of medium basic sites, accompanied by an enhancement in the strength of weak basic sites. In the strong basic site range (>500 °C), the CO_2_ desorption signals of all LDO catalysts showed an upward trend, among which MgAl-LDO and Ni-LDO exhibited weaker CO_2_ desorption peaks. Notably, the Co-LDO catalyst presented a sharp desorption peak around 600 °C, indicating that the introduction of the Co element could significantly enhance the strength of the strong basic sites of the catalyst.

The morphology and element distribution of the Co-LDO catalyst were observed by TEM and energy-dispersive X-ray spectroscopy (EDS) mapping. As shown in Figure 2g, Co-LDO exhibited a typical flake-like porous structure inherited from the LDH precursor, and no obvious metal agglomeration was observed. This observation was further confirmed by EDS mapping, which demonstrated the homogeneous distribution of Co, Mg, and Al elements within the catalyst. Consistent with the XRD and nitrogen adsorption–desorption results, Co was successfully doped into the MgAl oxide matrix to form a homogeneous lattice—a feature that was also distinguishable in the TEM images at a resolution of 5 nm.

### 3.2. Structure–Activity Relationship Between Catalyst Properties and OCF Performance

#### 3.2.1. Exploration and Optimization of Lignin Degradation Conditions

Within LDO catalytic systems, lignin DCM oil serves as the most critical indicator for evaluating the performance of the catalytic system [39,40]. To accurately determine the mass fraction of lignin DCM oil, the chemical composition of the poplar was measured: the cellulose content was 49.12 wt%, the hemicellulose content was 20.25 wt%, the lignin content was 27.93 wt%, and other components represented 2.7 wt%. To investigate the effects of the Co-LDO catalyst on the OCF performance of poplar, poplar powder was used as a substrate, and several key parameters, including the reaction temperature, time, air pressure, catalyst dosage, solvent type, and cobalt molar ratio in the LDO catalyst, were systematically investigated for their influences on the lignin DCM oil yield.

**Figure 3:**
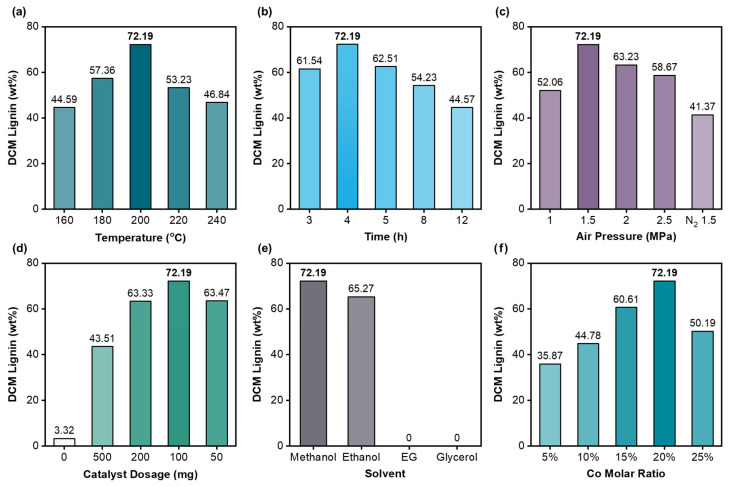
The yields of lignin DCM oil (wt%) with different conditions: (**a**) temperature; (**b**) time; (**c**) air pressure; (**d**) catalyst dosage; (**e**) solvent; (**f**) Co molar ratio.

As shown in Figure 3a, the yield of lignin DCM oil increased from 44.59 wt% to 72.19 wt% as the reaction temperature was elevated within the range of 160–200 °C. However, further increasing the temperature to 240 °C led to a decreased yield of 46.84 wt%, which was presumably attributed to the oxidation and degradation of lignin fragments into smaller molecules at higher temperatures [29]. Regarding the effect of the reaction time (Figure 3b), the maximum yield of lignin DCM oil was achieved at 4 h. Notably, a decrease in yield was observed with the further extension of the reaction time, accompanied by a reduction in the amount of solid residue collected from the reactor. This phenomenon was attributed not only to the overoxidation and degradation of lignin-derived small molecules but also to the overoxidation of cellulose and hemicellulose contained in the poplar powder. Subsequently, as Figure 3c illustrates, the influence of the system pressure was investigated under an air atmosphere. The pressure was maintained within the range of 1–2.5 MPa, and the maximum yield of lignin DCM oil (72.19 wt%) was attained at 1.5 MPa. Reduced yields were observed at lower pressures, which was ascribed to insufficient oxidative capacity for effective lignin depolymerization. Conversely, the excessive degradation of lignin was induced at elevated pressures due to intensified oxidative performance [28]. Under a nitrogen atmosphere, the partial depolymerization of lignin was observed; however, significantly lower lignin DCM oil yields were obtained in these control experiments due to the absence of oxygen, which compromised the oxidative depolymerization capacity.

Owing to the inherent alkalinity of Co-LDO catalysts, catalyst loading was investigated across a gradient range (Figure 3d). An excessive catalyst dosage was found to diminish the lignin oil yield due to pronounced alkalinity-induced side reactions. Conversely, an insufficient catalyst dosage resulted in inadequate depolymerization efficiency, attributable to suboptimal alkalinity levels. The near-complete absence of lignin DCM oil was recorded in the control experiments lacking Co-LDO, collectively confirming its essential catalytic role. Solvent effects were briefly evaluated in this catalytic system, as shown in Figure 3e. Consistent with literature reports, methanol and ethanol were identified as effective solvents for lignin dissolution, demonstrating competent catalytic performance within the OCF system, with respective yields of 72.19 wt% and 65.27 wt%. In contrast, ethylene glycol and glycerol were found to be catalytically inactive in the OCF process. Although these solvents enabled the extraction of lignin from poplar powder, which was rich in lignin–carbohydrate complex (LCC) bonds, they failed to facilitate depolymerization into lower-molecular-weight oligomers. Water was deliberately excluded as a solvent due to its propensity to induce the structural degradation of LDO catalysts [24]. As shown in Figure 3f, catalytic performance was evaluated across Co-LDO catalysts with varying cobalt molar ratios. The structural advantage was reflected in superior lignin degradation efficiency. The catalyst with 5% Co incorporation exhibited performance nearly identical to that of MgAl-LDO, while excessive Co loading (>20%) significantly reduced the lignin DCM oil yield. Collectively, these findings support two critical conclusions: the alkalinity introduced by Co species is essential for the effective oxidative catalytic fractionation of lignin; catalytic performance was constrained by threshold effects, and surpassing the optimal alkalinity or oxidation intensity diminishes the lignin oil yield through overdegradation.

#### 3.2.2. Comparing the Poplar Conversion Efficiency of Different Metal-Doped LDOs

**Figure 4:**
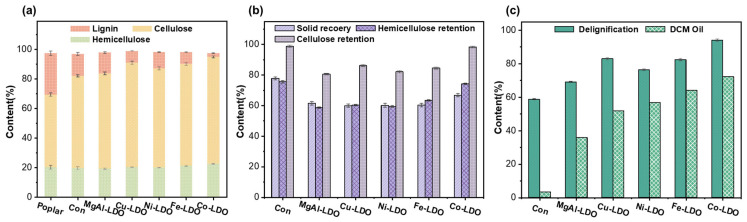
The chemical compositions (wt%) of (**a**) the original and OCF-treated substrates; (**b**) the solid yield, hemicellulose removal, and cellulose retention (wt%); (**c**) the delignification ratio and lignin DCM oil (wt%).

To investigate the impacts of different LDO catalysts on the OCF of poplar lignin, undoped MgAl-LDO, Cu-LDO, Ni-LDO, Fe-LDO, and Co-LDO catalysts were comparatively screened under optimal conditions [37]. As demonstrated by this study, the catalytic performance of OCF serves as a crucial consideration when developing novel catalytic systems for application in the lignin-first strategy. This study systematically elucidated the regulatory role of LDO catalysts doped with different metals in the catalytic oxidation fractionation of poplar biomass components, and it systematically investigated their catalytic performance under methanol-mediated oxidation conditions. Furthermore, the comprehensive chemical characterization of the catalytically treated poplar biomass residue was conducted, with the comparative results for component content presented in Figure 4.

The designed LDO catalytic systems featuring different metals all demonstrated superior catalytic decomposition effects on poplar biomass, as illustrated in Figure 4a. A significant increase in the relative content of cellulose and hemicellulose was observed in the residual matrix following OCF catalysis, accompanied by a considerable reduction in lignin content. This was mainly due to the excellent performance of the prepared LDO catalyst, which was able to retain cellulose and hemicellulose while removing lignin. In general, the cellulose retention rate, hemicellulose retention rate, delignification rate, and lignin DCM oil yield serve as key indicators for evaluating catalytic efficiency. Figure 4b illustrates the solid residue, cellulose, and hemicellulose retention ratios of poplar wood following catalysis, reflecting the retention of carbohydrates in poplar wood by the LDO-OCF catalytic system. Collectively, compared to the catalyst-free control group (Con), the reduction in solid residue following the OCF process using all LDO catalysts ranged from 59.91 wt% (Cu-LDO) to 66.70 wt% (Co-LDO). During this period, the reduction in solid content was primarily attributable to the removal of poplar lignin, alongside a minor decrease in carbohydrates. Although the control group maintained high retention rates of cellulose and hemicellulose, its delignification efficiency was only 58.70 wt%, failing to achieve high-efficiency removal. As shown in Figure 4c, the lignin DCM oil yield after reaction without LDO catalysts was also minimal at 3.32 wt%. Interestingly, both the delignification rates and DCM oil yields were significantly higher in systems catalyzed by LDOs, ranging from 69.06 wt% (MgAl-LDO) to 94.01 wt% (Co-LDO) and from 35.87 wt% (MgAl-LDO) to 72.19 wt% (Co-LDO). This indicates that the LDO catalytic system could enhance the OCF reaction performance of poplar, effectively cleaving lignin macromolecules into smaller-molecular-weight oligomers [41].

Overall, the cellulose and hemicellulose retention rates exhibited similar trends to the delignification rate. The LDO catalysts possessed specific Lewis basicity in the organic phase, and hemicellulose remained largely intact in the non-aqueous alkaline system [28]. This facilitated delignification and oxidation to form oligomers, while effectively protecting cellulose and hemicellulose from degradation. The solid recovery did not retain a similar trend in the MgAl-LDO control group due to its relatively moderate delignification efficiency (69.06 wt%), resulting in substantial lignin remaining in the solid residue [42]. In contrast, the Cu-LDO group exhibited superior delignification efficiency (82.93 wt%), leading to lower solid retention. The relative composition of the solid residue is illustrated in Figure 4a. Although the Cu-LDO group achieved higher lignin removal rates while retaining more hemicellulose, it produced less lignin DCM oil. This behavior was attributed to Cu-LDO exhibiting weaker bond-breaking activity under oxidative conditions, consistent with its characteristics as a Lewis acid. In comparison, the Co-LDO system demonstrated the most effective catalytic performance under identical reaction conditions, yielding the most favorable results. It achieved the highest solid retention rate (66.70 wt%), cellulose retention rate (98.23 wt%), hemicellulose retention rate (74.14 wt%), and delignification rate (94.01 wt%), with the converted lignin DCM oil also being high (72.19 wt%). The carbohydrates derived from OCF are suitable for the production of glucose, ethanol, and other high-value chemical derivatives. Determining the optimal OCF treatment conditions for poplar wood requires the comprehensive consideration of multiple dimensions (hemicellulose structure, lignin DCM oil component analysis, lignin structural changes), which will be systematically and thoroughly discussed and analyzed in the subsequent sections [40].

### 3.3. Structural Characterization of Carbohydrates Obtained After OCF

**Figure 5:**
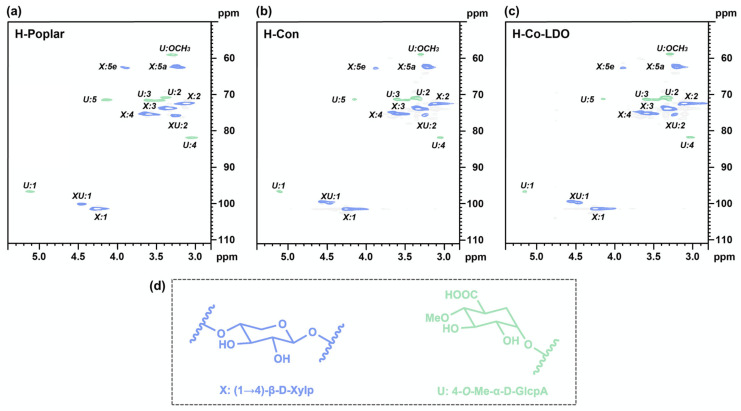
Structural characterization (2D-HSQC NMR spectra) of (**a**) original hemicellulose of poplar; hemicellulose extracted after OCF of poplar (**b**) without and (**c**) with Co-LDO; and (**d**) the identified main structures of hemicellulose.

Based on the lignin-first strategy, both reductive catalytic fractionation and oxidative catalytic fractionation methods inevitably lead to the degradation of the carbohydrate fraction [18]. Therefore, preventing changes in hemicellulose’s structural integrity efficiently while achieving the high-efficiency catalytic degradation of biomass has become a critical challenge in this field [13,18]. To comprehensively investigate the structural preservation characteristics of hemicellulose under optimal process conditions (Co-LDO, 200 °C, 4 h, air 1.5 MPa, methanol), the structural changes in hemicellulose before and after OCF were characterized by 2D-HSQC NMR [39,42]. Poplar hemicellulose is mainly composed of a (1→4)-linked *β*-D-xylopyranose (Xylp) backbone and 4-*O*-methyl-D-glucuronic acid (MeGlcA) side chains, and the characteristic chemical shifts are mainly distributed in the range of δ_C_/δ_H_ 50–111/2.8–5.4. As shown in Figure 5, the hemicellulose extracted from raw poplar (H-Poplar) showed clear characteristic cross-peaks of xylose units: X1 (δ_C_/δ_H_ 102.1/4.32), X2 (δ_C_/δ_H_ 73.2/3.08), X3 (δ_C_/δ_H_ 74.5/3.21), X4 (δ_C_/δ_H_ 76.3/3.52), X5a (δ_C_/δ_H_ 63.2/3.92), and X5e (δ_C_/δ_H_ 65.8/3.28). The characteristic cross-peaks of MeGlcA units (U1–U5) were also clearly observed.

Notably, the hemicellulose extracted from Co-LDO-catalyzed OCF residues (H-Co-LDO) showed almost the same cross-peak positions, shapes, and intensities as H-Poplar; in particular, the characteristic peaks of xylose backbone units remained almost unchanged. This indicated that the xylose backbone structure of hemicellulose was well preserved during the OCF process, and the glycosidic bonds between xylose units were not cleaved in large quantities. The hemicellulose extracted from the catalyst-free control group (H-Con) also showed similar spectral characteristics to H-Poplar, which further confirms that the non-alkaline methanol system itself would not cause the significant degradation of hemicellulose. In both the H-Co-LDO and H-Con spectra, the intensity of the characteristic peaks of MeGlcA units (U1–U5) decreased slightly compared with H-Poplar. This indicates that methanol as a reaction solvent promotes the degradation of a small amount of MeGlcA side chains, leading to the partial structural damage of hemicellulose. At the same time, the xylose backbone structure remained relatively intact, which contrasts sharply with the situation in traditional OCF processes, where the hemicellulose backbone is extensively degraded and broken down into small organic molecules.

The above observation was further supported by GPC analysis, in which the molecular weights of H-Poplar, H-Con, and H-Co-LDO were also measured (Table S6). The molecular weights of H-Poplar were 107,940 g/mol (M_w_) and 38,460 g/mol (M_n_), with a PDI of 2.81. The M_w_ values for the catalyst-free H-Con control group and the experimental H-Co-LDO group were 89,640 g/mol and 86,930 g/mol, with M_n_ values of 32,520 g/mol and 31,570 g/mol and PDI values of 2.76 and 2.75, respectively. In the methanol system, the M_w_ and M_n_ of H-Con were slightly lower than those of H-Poplar, which is unavoidable in high-temperature reactions; however, the H-Co-LDO obtained after adding the Co-LDO catalyst showed only a slight decrease compared to the catalyst-free control group. This also indicates that the degradation of hemicellulose could be effectively prevented in the Co-LDO OCF reaction, while cellulose retention reached 98.23 wt%. Co-LDO achieved the high retention of carbohydrates in the OCF reaction system.

Combined with the catalyst characterization results, the retention mechanism of hemicellulose in this Co-LDO-catalyzed non-alkaline OCF system was revealed: (1) the additive-free non-alkaline system avoids the strong alkaline hydrolysis of hemicellulose’s glycosidic bonds in traditional alkaline OCF systems; (2) the moderate basicity of the Co-LDO catalyst is mainly used to activate lignin linkages, and it will not cause a large amount of glycosidic bond cleavage; (3) the Co-LDO catalyst further reduces the likelihood of hemicellulose and cellulose degradation, thereby effectively preserving the carbohydrates. In summary, the Co-LDO catalyst designed in this study for the poplar OCF process achieved the effective preservation of the structural integrity of hemicellulose components under the lignin-first degradation strategy. The proposed method not only effectively avoided the drawback of excessive hemicellulose degradation in the traditional lignin-first catalytic strategy, but also provides high-quality raw material support for the downstream high-value application of biomass refining.

### 3.4. Structural Characterization and Degradation Mechanism of Lignin After OCF

#### 3.4.1. Molecular Weight and Monomer Composition of Lignin DCM Oil

**Figure 6:**
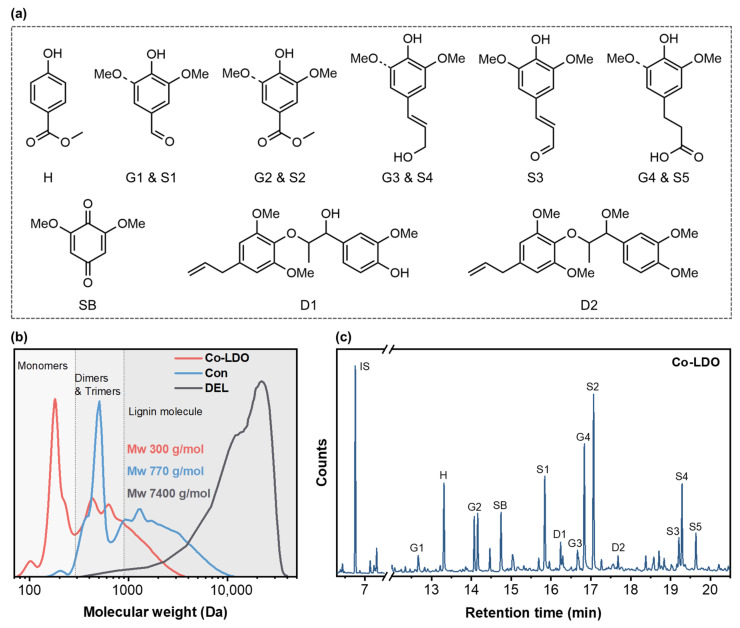
Structural characterization of lignin DCM oil: (**a**) types of phenolic oligomers; (**b**) molecular weight; (**c**) GC-MS spectrum.

Lignin is the main component of biomass, and its high-value utilization is a critical prerequisite for the efficient development of biomass refining; it directly determines the comprehensive efficiency of the whole process [43,44]. In the LDO catalytic system, the generation of low-molecular-weight lignin oligomeric oil is the core criterion for evaluating the catalytic depolymerization efficiency of biomass, and its molecular weight and chemical composition are both key evaluation indicators [45]. The yields of lignin DCM oil obtained from the OCF reaction of poplar catalyzed by different metal-doped LDO catalysts were quantitatively determined, which confirmed that the Co-LDO catalyst exhibited the optimal catalytic efficiency. To investigate the effects of the Co-LDO catalytic system on the molecular weight of lignin, gel permeation chromatography (GPC) was adopted to determine the relative molecular weights of lignin products. As shown in Figure 6b, the weight-average molecular weight (M_w_) of the native lignin (DEL) polymer extracted from poplar was 7400 g/mol, and its molecular weight distribution was mainly concentrated in the lignin polymer range. The M_w_ of lignin oil obtained from the blank reaction without a catalyst (Con) was 770 g/mol, and its molecular weight distribution shifted significantly to the low-molecular-weight range. The product contained partial lignin monomers and oligomers (dimers, trimers, M_w_ 120–1000 g/mol), with a large amount of high-molecular-weight polymers (M_w_ > 1000 g/mol) still existing simultaneously. After the addition of the Co-LDO catalyst, the M_w_ of the obtained lignin DCM oil (Co-LDO under air) decreased significantly to 300 g/mol. The product was dominated by lignin oligomers, in which dimers and trimers accounted for the largest proportion, with fewer lignin monomers and only a small amount of lignin high-molecular-weight polymers.

Methanol was used as the solvent, and the oxidative catalytic fractionation reaction of poplar was carried out under the action of the Co-LDO catalyst. The soluble lignin fraction (lignin DCM oil) was obtained by extraction with dichloromethane. GC-MS and GC-FID technologies were adopted to conduct qualitative identification and quantitative analysis on the phenolic monomers in this fraction [35]. The results in Figure 6a,c illustrate that lignin underwent oxidation and bond cleavage with the action of the Co-LDO catalyst, the types and distribution characteristics of the generated phenolic monomers were clear, and only trace amounts of dimers were detected. Due to the complex composition of the oil derived from lignin oxidation products, which include minor peaks of carbohydrates, this study systematically analyzed only the phenolic monomers associated with lignin sources. The quantitative results of GC-FID indicated that the total content of all lignin-derived phenolic monomers accounted for approximately 9 wt% of the total lignin content in poplar. GC-MS analysis showed that *p*-coumaryl alcohol lignin (H-type) was only oxidized to generate H phenolic monomers with a methyl formate structure in the side chain. Notably, the monomer products generated by coniferyl alcohol lignin (G-type) and sinapyl alcohol lignin (S-type) presented significant regularity. When the side chain contained one carbon atom, G_1_, S_1_, G_2_, and S_2_ monomers were generated, among which the content of the S_2_ monomer was the highest, followed by the S_1_ monomer, and the difference between the two was related to the aldehyde group methyl esterification induced by methanol as the reaction solvent. The G_1_ and G_2_ monomers also presented similar content distribution characteristics, and the G_2_ monomer had isomers, which showed two characteristic peaks in the GC-MS spectrum. Lignin phenolic monomers with propyl side chains included G_3_, G_4_, S_3_, S_4_, and S_5_; no methyl esterification reaction occurred in such monomers, and the 2-propenyl groups and terminal oxygen-containing groups contained in them could form a stable p-π-conjugated system [46]. In addition, propyl phenolic monomers could be further oxidized to generate propionyl-substituted G_4_ and S_5_ monomers, the content of the G_4_ monomer was higher than that of the S_5_ monomer, and such phenolic monomers did not undergo a methyl esterification reaction in the methanol system. Meanwhile, quinone monomer SB and trace amounts of D_1_ and D_2_ dimers were detected, the α-position hydroxyl groups of these dimers underwent a methanol methoxylation reaction, and the terminal propyl groups were oxidized to 3-propenyl groups. In conclusion, the Co-LDO catalytic system can significantly reduce the molecular weight of poplar lignin while yielding phenolic monomers and oligomers, thereby presenting significant potential for developing downstream lignin-based high-value derivatives [12].

#### 3.4.2. Structural Variation in Lignin Characterized by 2D-HSQC NMR

**Figure 7:**
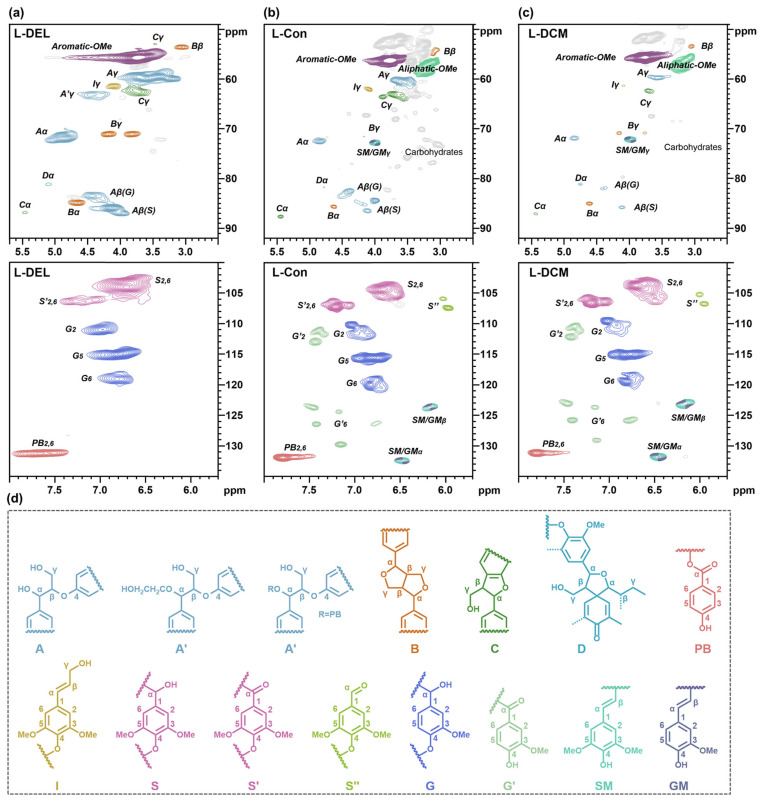
Structural characterization (2D-HSQC NMR spectra) of (**a**) poplar DEL lignin; lignin DCM oil in the OCF of poplar (**b**) without and (**c**) with Co-LDO; and (**d**) the identified main structures of the lignin side chain and aromatic regions.

To elucidate the structural differences between poplar lignin and lignin DCM oil, as well as the catalytic mechanism of the Co-LDO catalyst in lignin bond cleavage, 2D-HSQC NMR spectroscopy was employed to systematically analyze the detailed structural characteristics of lignin [47]. In the 2D-HSQC spectra (Figure 7) of three lignin components (L-DEL, L-Con, L-DCM), characteristic signals of typical bonds (β-*O*-4, β-5, β-β) and lignin aromatic units were localized in distinct chemical shift regions. Specifically, these regions encompassed the characteristic side-chain region (δ_C_/δ_H_, 49–92/2.5–5.7) and the aromatic region (δ_C_/δ_H_, 100–135/5.7–8.0) [9]. The side-chain region clearly revealed inter-unit bonding and structural variations, while the aromatic region aided in confirming the type of aromatic monomer. Combining NMR and GC-MS spectra, the mechanism of lignin bond cleavage during the conversion of L-DEL to L-DCM was elucidated. Notably, the 2D HSQC NMR spectra of methanol-treated samples (L-Con, Figure 7b) and cobalt LDO-catalyzed samples (L-DCM, Figure 7c) exhibited significant differences compared to native poplar lignin (L-DEL, Figure 7a), indicating the substantial catalytic oxidative bond cleavage of poplar lignin during the OCF reaction.

In the side-chain region of native poplar lignin (L-DEL, Figure 7a), strong characteristic cross-peaks of β-*O*-4 aryl ether linkages (A_α_, A_β_, A_γ_), β-5 phenylcoumaran linkages (B_α_, B_β_, B_γ_) and β-β resinol linkages (C_γ_) were clearly observed. After methanol treatment without a catalyst (L-Con, Figure 7b), the intensity of these cross-peaks decreased significantly, indicating that the partial cleavage of lignin linkages occurred under thermal oxidation. After the introduction of the Co-LDO catalyst, the characteristic signals of β-*O*-4 linkages in lignin almost completely disappeared [47]. A small amount of characteristic signals of A-type structures still remained in the lignin oil, which was attributed to the presence of D_1_, D_2_ dimers and other oligomer fractions in L-Con and L-DCM. Meanwhile, the intensities of the characteristic signals for B-type side-chain moieties (B_α_, B_β_, B_γ_) and C-type side-chain moieties (C_γ_) were also significantly reduced, which confirmed that the Co-LDO catalyst could efficiently cleave the β-β and β-5 linkages in lignin. The overall reduction in the signals related to the above two types of C-O and C-C linkages indicated that the methanol solvent and Co-LDO catalyst could synergistically catalyze the oxidative degradation and depolymerization of lignin into smaller molecules. Notably, characteristic signals of certain carbohydrates were detectable in L-Con oil without dichloromethane extraction, which further confirmed that the primary components in the L-DCM oil originated from lignin. The aromatic region in the 2D-HSQC spectrum clearly reveals the types of lignin structural units and can distinctly differentiate the structural features of aromatic monomers bearing different para-substituted groups [10,47]. As indicated by the spectrum, the appearance of new G′_2_, G′_6_, and S″ signals confirmed the presence of G_1_, S_1_, G_2_, and S_2_ monomers; the disappearance of the A′_γ_ signal and the residual PB′_2,6_ signals corroborated the existence of H monomers. Meanwhile, the presence of aliphatic OME signals indicated the methyl esterification of G_1_ and S_2_ monomers, consistent with the aforementioned inferences. Additionally, the detection of SM and GM_α_ signals verified the presence of G_3_, S_3_, and S_4_ aromatic monomers in the lignin oil. Collectively, the lignin phenolic linkages and monomer species identified via the 2D-HSQC spectra are fully consistent with the GC-MS spectral signals, further confirming that the Co-LDO catalyst exhibited superior catalytic activity toward the cleavage of C-O and C-C linkages in lignin.

### 3.5. Mass Balance

**Figure 8:**
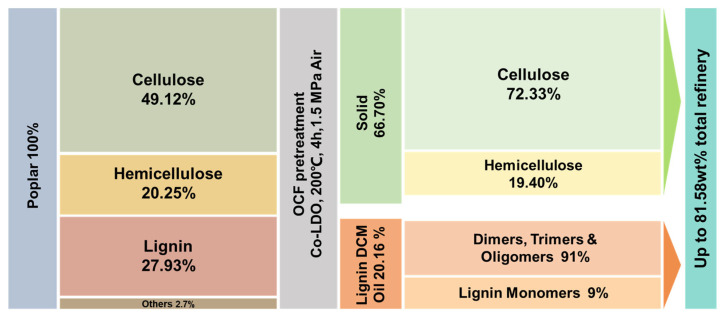
Mass balance of the non-alkaline Co-LDO catalyst system.

Based on the preceding results, the optimal catalytic conditions for oxidative catalytic fractionation (Co-LDO, 200 °C, 4 h, air 1.5 MPa, methanol solvent) were confirmed as the most effective for poplar deconstruction. This process efficiently retained the majority of structurally intact hemicellulose and nearly all cellulose, while effectively removing lignin and converting it into monomers and oligomers. Figure 8 displays the relative mass balance calculated after conducting experiments in a 2 L reactor following the 40-times scale-up of the Co-LDO oxidative catalytic distillation strategy for poplar wood under optimal conditions. Following catalytic refining, the catalytic residue constituted 66.70% of the total mass, comprising 72.33% cellulose and 19.40% hemicellulose. Concurrently, the lignin catalytic fraction accounted for 20.16% of the lignin DCM oil, containing approximately 9% lignin monomers and 91% lignin oligomers. In summary, under optimal oxidative catalytic fractionation conditions, the proposed lignin-first OCF strategy achieved a total utilization rate of lignocellulose components that was as high as 81.58%. Specifically, it efficiently preserved hemicellulose with well-maintained structural integrity from catalytic degradation into small molecules, while the catalytic conversion of lignin yielded lignin oligomer oil. Therefore, the Co-LDO catalyst prepared by this simple method represents an ideal catalytic conversion technology for the effective deconstruction and value-added processing of poplar lignocellulose biomass under additive-free non-alkaline conditions.

## 4. Conclusions

In this work, a Co-doped layered double oxide (Co-LDO) catalyst was rationally designed and synthesized, and an additive-free non-alkaline (without Brønsted bases) OCF system was developed for the lignin-first full-component refining of poplar biomass. This system overcame the core bottleneck of excessive carbohydrate degradation in traditional OCF processes and achieved efficient delignification, the high-value conversion of lignin, and the high retention of carbohydrates simultaneously. The results indicate that the Co-LDO catalytic system demonstrates excellent catalytic performance, with a delignification rate of up to 94.01 wt% and with the carbohydrate residue retaining 98.23 wt% cellulose and 74.14 wt% hemicellulose (M_w_ = 86,830 g/mol, M_n_ = 31,570 g/mol). Moreover, 72.19 wt% of lignin was converted into lignin DCM oil (M_w_ = 300 g/mol). Under optimal conditions (Co-LDO, 200 °C, 4 h, 1.5 MPa air, methanol solvent), the Co-LDO-catalyzed OCF system achieved over 90% total carbohydrate retention and 81.58% total refining efficiency. In summary, the Co-LDO catalyst proposed for the lignin-first OCF strategy demonstrates high activity as a non-precious metal catalyst; it not only yields highly reactive lignin but also enables the solid recovery of carbohydrates, thereby providing important theoretical and technical support for the development of lignin-first biorefining strategies.

## Data Availability

The original contributions presented in this study are included in the article/Supplementary Materials. Further inquiries can be directed to the corresponding author.

## Supplementary Materials

The following supporting information can be downloaded at: https://www.mdpi.com/article/10.3390/polym18080922/s1, Section S1. Material and methods; Table S1. Lignin DCM oil yield under OCF different conditions; Table S2. BET surface area (m^2^/g), Pore diameter (nm) of Co-LDO with different Co doping ratios; Table S3. Chemical composition (Cellulose, Hemicellulose, Lignin) of the different LDOs; Table S4. Solid yield, Delignification, Hemicellulose retention, Cellulose retention, Lignin DCM oil (wt%) of the different LDOs; Table S5. Assignments (ppm) of ^13^C-^1^H cross signals in 2D-HSQC NMR spectra of hemicellulose fractions; Table S6. Molecular weights of the hemicelluloses; Table S7. Assignments (ppm) of ^13^C-^1^H cross signals in 2D-HSQC NMR spectra of lignin fractions.

## References

[1] Ragauskas A.J., Williams C.K., Davison B.H., Britovsek G., Cairney J., Eckert C.A., Frederick W.J., Hallett J.P., Leak D.J., Liotta C.L. (2006). The path forward for biofuels and biomaterials. Science.

[2] Sun Z., Fridrich B., de Santi A., Elangovan S., Barta K. (2018). Bright side of lignin depolymerization: Toward new platform chemicals. Chem. Rev..

[3] Wang W., Zhou Y., Zhu J., Jin Y., Chen J., Liu C., Ma J., Nie X., Wang P., Wen Y. (2025). Synergistic effect between Fe and Ni cations of LaFe_1-x_Ni_x_O_3_ perovskites enables efficient catalytic wet aerobic oxidation of corncob alkali lignin in base-free system to produce valuable aromatic aldehydes and acids. Chem. Eng. J..

[4] Alonso D.M., Hakim S.H., Zhou S., Won W., Hosseinaei O., Tao J., Garcia-Negron V., Motagamwala A.H., Mellmer M.A., Huang K. (2017). Increasing the revenue from lignocellulosic biomass: Maximizing feedstock utilization. Sci. Adv..

[5] Calcio Gaudino E., Cravotto G., Manzoli M., Tabasso S. (2021). Sono- and mechanochemical technologies in the catalytic conversion of biomass. Chem. Soc. Rev..

[6] Shuai L., Amiri M.T., Questell-Santiago Y.M., Heroguel F., Li Y., Kim H., Meilan R., Chapple C., Ralph J., Luterbacher J.S. (2016). Formaldehyde stabilization facilitates lignin monomer production during biomass depolymerization. Science.

[7] Abu-Omar M.M., Barta K., Beckham G.T., Luterbacher J.S., Ralph J., Rinaldi R., Román-Leshkov Y., Samec J.S.M., Sels B.F., Wang F. (2021). Guidelines for performing lignin-first biorefining. Energy Environ. Sci..

[8] Bartling A.W., Stone M.L., Hanes R.J., Bhatt A., Zhang Y., Biddy M.J., Davis R., Kruger J.S., Thornburg N.E., Luterbacher J.S. (2021). Techno-economic analysis and life cycle assessment of a biorefinery utilizing reductive catalytic fractionation. Energy Environ. Sci..

[9] Zhang H., Sun Q., Liu Y.-B., Ma C.-Y., Wen J.-L., Li Z., Yuan T.-Q. (2026). Green and efficient alkaline DES pretreatment for full-component fractionation of reed biomass: Hemicelluloses, uncondensed lignin and easily digestible cellulose. Chem. Eng. J..

[10] Meng G., Lan W., Zhang L., Wang S., Zhang T., Zhang S., Xu M., Wang Y., Zhang J., Yue F. (2023). Synergy of single atoms and lewis acid sites for efficient and selective lignin disassembly into monolignol derivatives. J. Am. Chem. Soc..

[11] Xiao L.-P., Wang S., Li H., Li Z., Shi Z.-J., Xiao L., Sun R.-C., Fang Y., Song G. (2017). Catalytic hydrogenolysis of lignins into phenolic compounds over carbon nanotube supported molybdenum oxide. ACS Catal..

[12] Zhang Z.-H., Wu X., Ren X., Rong Z., Sun Z., Barta K., Yuan T.-Q. (2023). High yield production of 1,4-cyclohexanediol from lignin derived 2,6-dimethoxybenzoquinone via Raney NiMn catalyst in hydrogen free conditions. J. Energy Chem..

[13] Van den Bosch S., Schutyser W., Vanholme R., Driessen T., Koelewijn S.F., Renders T., De Meester B., Huijgen W.J.J., Dehaen W., Courtin C.M. (2015). Reductive lignocellulose fractionation into soluble lignin-derived phenolic monomers and dimers and processable carbohydrate pulps. Energy Environ. Sci..

[14] Zhang J., Webber M.S., Pu Y., Li Z., Meng X., Stone M.L., Wei B., Wang X., Yuan S., Klein B. (2025). Sustainable aviation fuels from biomass and biowaste via bio- and chemo-catalytic conversion: Catalysis, process challenges, and opportunities. Green Energy Environ..

[15] González D., Santos V., Parajó J.C. (2011). Manufacture of fibrous reinforcements for biocomposites and hemicellulosic oligomers from bamboo. Chem. Eng. J..

[16] Schutyser W., Renders T., Van den Bosch S., Koelewijn S.F., Beckham G.T., Sels B.F. (2018). Chemicals from lignin: An interplay of lignocellulose fractionation, depolymerisation, and upgrading. Chem. Soc. Rev..

[17] Huang X., Morales Gonzalez O.M., Zhu J., Korányi T.I., Boot M.D., Hensen E.J.M. (2017). Reductive fractionation of woody biomass into lignin monomers and cellulose by tandem metal triflate and Pd/C catalysis. Green Chem..

[18] Zhu Y., Liao Y., Lu L., Lv W., Liu J., Song X., Wu J., Li L., Wang C., Ma L. (2023). Oxidative catalytic fractionation of lignocellulose to high-yield aromatic aldehyde monomers and pure cellulose. ACS Catal..

[19] Schutyser W., Kruger J.S., Robinson A.M., Katahira R., Brandner D.G., Cleveland N.S., Mittal A., Peterson D.J., Meilan R., Román-Leshkov Y. (2018). Revisiting alkaline aerobic lignin oxidation. Green Chem..

[20] Zirbes M., Quadri L.L., Breiner M., Stenglein A., Bomm A., Schade W., Waldvogel S.R. (2020). high-temperature electrolysis of kraft lignin for selective vanillin formation. ACS Sustain. Chem. Eng..

[21] Zhu Y., Liao Y., Lv W., Liu J., Song X., Chen L., Wang C., Sels B.F., Ma L. (2020). Complementing vanillin and cellulose production by oxidation of lignocellulose with stirring control. ACS Sustain. Chem. Eng..

[22] Liu M., Dyson P.J. (2023). Direct conversion of lignin to functionalized diaryl ethers via oxidative cross-coupling. Nat. Commun..

[23] Langis-Barsetti S., Beaudoin D., Palus E., Gagné A. (2025). High-yield production of aromatic aldehydes from lignin via oxidative depolymerization. ACS Sustain. Chem. Eng..

[24] Sun Z., Bottari G., Afanasenko A., Stuart M.C.A., Deuss P.J., Fridrich B., Barta K. (2018). Complete lignocellulose conversion with integrated catalyst recycling yielding valuable aromatics and fuels. Nat. Catal..

[25] Peng F., Ren J.-L., Xu F., Bian J., Peng P., Sun R.-C. (2009). Comparative study of hemicelluloses obtained by graded ethanol precipitation from sugarcane bagasse. J. Agric. Food Chem..

[26] Li C., Zhao X., Wang A., Huber G.W., Zhang T. (2015). Catalytic transformation of lignin for the production of chemicals and fuels. Chem. Rev..

[27] Luo Z., Liu C., Radu A., de Waard D.F., Wang Y., Behaghel de Bueren J.T., Kouris P.D., Boot M.D., Xiao J., Zhang H. (2024). Carbon–carbon bond cleavage for a lignin refinery. Nat. Chem. Eng..

[28] Luo H., Weeda E.P., Alherech M., Anson C.W., Karlen S.D., Cui Y., Foster C.E., Stahl S.S. (2021). oxidative catalytic fractionation of lignocellulosic biomass under non-alkaline conditions. J. Am. Chem. Soc..

[29] Renders T., Schutyser W., Van den Bosch S., Koelewijn S.-F., Vangeel T., Courtin C.M., Sels B.F. (2016). influence of acidic (H_3_PO_4_) and alkaline (NaOH) additives on the catalytic reductive fractionation of lignocellulose. ACS Catal..

[30] Zhou Y., Xiong W., Jin Y., Wang P., Wei W., Ma J., Zhang X. (2023). Catalytic aerobic oxidation of lignin-based vanillyl alcohol under base-free conditions over an efficient and reusable LaFeO3 perovskite for vanillin production. Green Chem..

[31] Du X., Tricker A.W., Yang W., Katahira R., Liu W., Kwok T.T., Gogoi P., Deng Y. (2021). Oxidative catalytic fractionation and depolymerization of lignin in a one-pot single-catalyst system. ACS Sustain. Chem. Eng..

[32] Cui Y., Weeda E.P., Omolabake S., Karlen S.D., Holland C.M., Stahl S.S. (2024). Oxidative catalytic fractionation of lignocellulosic biomass using a Co-N-P-C catalyst and one-step isolation of aromatic monomers via centrifugal partition chromatography. ACS Sustain. Chem. Eng..

[33] Klein I., Marcum C., Kenttämaa H., Abu-Omar M.M. (2016). Mechanistic investigation of the Zn/Pd/C catalyzed cleavage and hydrodeoxygenation of lignin. Green Chem..

[34] Rinesch T., Bolm C. (2018). Cobalt-Catalyzed Oxidation of the β-O-4 bond in lignin and lignin model compounds. ACS Omega.

[35] Gu N.X., Palumbo C.T., Bleem A.C., Sullivan K.P., Haugen S.J., Woodworth S.P., Ramirez K.J., Kenny J.K., Stanley L.D., Katahira R. (2023). Autoxidation catalysis for carbon-carbon bond cleavage in lignin. ACS Cent. Sci..

[36] Gao D., Ouyang D., Zhao X. (2024). Controllable oxidative depolymerization of lignin to produce aromatic aldehydes and generate electricity under mild conditions with direct biomass fuel cells as flexible reactors. Chem. Eng. J..

[37] Huo L., Wang T., Pu Y., Li C., Li L., Zhai M., Qiao C., Bai Y. (2021). Effect of Cobalt doping on the stability of CaO-based catalysts for dimethyl carbonate synthesis via the transesterification of propylene carbonate with methanol. ChemistrySelect.

[38] Lv W.-L., He L., Li W.-C., Zhou B.-C., Lv S.-P., Lu A.-H. (2023). Atomically dispersed Co^2+^ on MgAlOx boosting C_4-10_ alcohols selectivity of ethanol valorization. Green Chem..

[39] Ma C.Y., Xu L.H., Zhang C., Guo K.N., Yuan T.Q., Wen J.L. (2021). A synergistic hydrothermal-deep eutectic solvent (DES) pretreatment for rapid fractionation and targeted valorization of hemicelluloses and cellulose from poplar wood. Bioresour. Technol..

[40] Sun Q., Wang B., Huang H., Ma C.-Y., Ma Y., Shen X., Cao X., Sun Z., Zhang L., Yuan T.-Q. (2024). Pressure-assisted hydrothermal pretreatment for biorefinery to enhance pulp production. Chem. Eng. J..

[41] Yuan Z., Klinger G.E., Nikafshar S., Cui Y., Fang Z., Alherech M., Goes S., Anson C., Singh S.K., Bals B. (2021). Effective biomass fractionation through oxygen-enhanced alkaline-oxidative pretreatment. ACS Sustain. Chem. Eng..

[42] Ma C.Y., Xu L.H., Sun Q., Sun S.N., Cao X.F., Wen J.L., Yuan T.Q. (2022). Ultrafast alkaline deep eutectic solvent pretreatment for enhancing enzymatic saccharification and lignin fractionation from industrial xylose residue. Bioresour. Technol..

[43] Andriani F., Lawoko M. (2024). Oxidative Carboxylation of Lignin: Exploring Reactivity of Different Lignin Types. Biomacromolecules.

[44] Yu X., Wei Z., Lu Z., Pei H., Wang H. (2019). Activation of lignin by selective oxidation: An emerging strategy for boosting lignin depolymerization to aromatics. Bioresour. Technol..

[45] Palumbo C.T., Gu N.X., Bleem A.C., Sullivan K.P., Katahira R., Stanley L.M., Kenny J.K., Ingraham M.A., Ramirez K.J., Haugen S.J. (2024). Catalytic carbon-carbon bond cleavage in lignin via manganese-zirconium-mediated autoxidation. Nat. Commun..

[46] Xu E., Xie F., Liu T., He J., Zhang Y. (2024). Photocatalytic, Oxidative cleavage of C-C bond in lignin models and native lignin. Chem.-Eur. J..

[47] Wen J.-L., Sun S.-L., Yuan T.-Q., Sun R.-C. (2015). Structural elucidation of whole lignin from eucalyptus based on preswelling and enzymatic hydrolysis. Green Chem..

