# Supplementary material for: Effective Deconstruction of Lignocellulose Through Oxidative Catalytic Fractionation Under Additive-Free Non-Alkaline System via Co-LDO Catalyst
Source: Polymers (Basel). 2026 Apr 9;18(8):922. doi: 10.3390/polym18080922 (PMC13119687; doi:10.3390/polym18080922)
Supplement: Supplementary file 1 [file polymers-18-00922-s001.zip › polymers-4224313-supplementary.pdf]

Supplementary information

Effective deconstruction of lignocellulose through oxidative catalytic fractionation under additive-free non-alkaline system via Co-LDO catalyst

Haozhi Zhang [1,2,3], Wei Yan [1], Ying Wang [1], Cheng-Ye Ma [2,3,*], Changfu Zhuang [1,*]

[1] *International Joint Research Center for Biomass Materials, Southwest Forestry University, Kunming 650051, China.*

[2] *Department of Biosystems Engineering, Zhejiang University, 866 Yuhangtang Road, Hangzhou, 310058, China*

[3] *Institute of Zhejiang University-Quzhou, 99 Zheda Road, Quzhou, 324000, China*

[*] *Corresponding author: chengye.ma@foxmail.com (C.-Y. Ma), cfzhuang@swfu.edu.cn (C.-F. Zhuang).*

Content

S1. Material and methods......3

S1.1 Characterization of catalysts......3

S1.2 Preparation of double enzymatic lignin (DEL)......4

S1.3 Acetylation of double enzymatic lignin (DEL)......4

S1.4 Separation of hemicellulose from poplar and OCF-treated residues......5

S1.5 Structure elucidation of the substrates after OCF......6

S1.6 Characterization of the lignin fractions......7

S1.7 Characterization of the hemicellulose......8

S2. Tables and Figures......10

Table S1. Lignin DCM oil yield under OCF different conditions......10

Table S2. BET surface area (m^2/g), Pore diameter (nm) of Co-LDO with different Co doping ratios......11

Table S3. Chemical composition (Cellulose, Hemicellulose, Lignin) of the different LDOs......12

Table S4. Solid yield, Delignification, Hemicellulose retention, Cellulose retention, Lignin DCM oil (wt%) of the different LDOs......13

Table S5. Assignments (ppm) of ^{13}C-^{1}H cross signals in 2D-HSQC NMR spectra of hemicellulose fractions......14

Table S6. Molecular weights of the hemicelluloses......15

Table S7. Assignments (ppm) of ^{13}C-^{1}H cross signals in 2D-HSQC NMR spectra of lignin fractions......16

S1. Material and methods

S1.1 Characterization of catalysts

X-ray diffraction (XRD) measurements were analysed on a Bruker D8 Advanced diffractometer equipped with Cu Kα at 30 kV and 30 mA. The scan rate was 5° in the 2θ range from 5° to 90°.

X-ray photoelectron spectroscopy (XPS) measurements were performed using a Thermo escalab 250 Xi spectrometer equipped with Al Ka radiation ($h\nu$ = 1486.6 eV) under ultrahigh vacuum. The binding energies were calibrated internally by carbon deposit C1 s with an E_b of 284.8 eV.

The specific surface areas and pore diameter of the catalysts were determined using a Kubo X1000 apparatus. The samples were first degassed at 200 °C for 3 h and then analysed via liquid nitrogen adsorption at -196 °C.

Temperature programmed desorption of CO_2 (CO_2-TPD) was carried out on a Altamira Instruments AMI-300 apparatus with a thermal conductivity detector for the measurement of the basicity of the catalysts. Each sample (0.15 g) was placed in a quartz U-tube reactor, pretreated under helium flow at 800 °C for 1 h, and then cooled to 50 °C under He flow. The adsorption of CO_2 was performed at 50 °C with a flow of 30 mL min^{-1} of 30% CO_2/He for 60 min, followed by a He purge for another 30 min to remove the physisorbed CO_2. The desorption process was performed at a heating rate of 10 °C/min from 50 °C to 800 °C under helium flow.

Transmission electron microscopy (TEM) with a JEM-F200 apparatus (200kV, JED-2300T) electron microscope operated at an acceleration voltage of 200 kV was conducted to obtain the general morphology of the catalysts.

S1.2 Preparation of double enzymatic lignin (DEL)

The ball-milled poplar powder (5 g) was mixed with the desired amounts of sodium acetate buffer (pH 4.8) with a solid-to-liquid ratio of 1:20 (g/mL) and cellulase (35 FPU/g substrate). Then the mixture was incubated at 50 °C in a rotary shaker with a rotational velocity of 150 rpm for 48 h. Next, the mixture was centrifuged and the residue was washed thoroughly with sodium acetate buffer (pH 4.8) to remove the hydrolyzed carbohydrates, and then freeze-dried. Finally, the dried residual solid was repeatedly subjected to ball-milling for 2 h and enzymatic hydrolysis again as above-mentioned processes. After washing with acidic water (pH 2.0) and freeze-drying, DEL sample was obtained. To increase the solubility of the lignin in tetrahydrofuran (THF) for the molecular weights determination by GPC technique, the acetylation of lignin was performed.

S1.3 Acetylation of double enzymatic lignin (DEL)

About 30 mg of dry lignin was dissolved in 3 mL of a solution of dimethyl sulfoxide: 1-methylimidazole (2:1, v/v) and stirred without direct light at room temperature for 12 h. Acetic anhydride (1.0 mL) was added to the reaction mixture and

continued reacting for 2 h. The reaction mixture was dropped slowly into 100 mL acid water (pH = 2) to induce precipitation followed by centrifugation, the acetylated lignin was obtained.

S1.4 Separation of hemicellulose from poplar and OCF-treated residues

Separate the catalyst from the biomass residue OCF-treated. Place 0.5 g of sample and 0.5 g of sodium chlorite in a conical flask, add 10 mL of water, and adjust the solution (pH 3.8-4.0) with acetic acid. After thorough mixing and sealing the solution, react at 75°C in a water bath for 1 h. Add a further 0.5 g sodium chlorite and an equal volume of acetic acid to adjust the pH, continuing the reaction for 1 h. Wash the residue repeatedly with clean water, then add 15 mL of 10% (w/w) potassium hydroxide solution. Stir at room temperature for 12 h. Collect the filtrate by vacuum filtration. Wash the residue three times with clean water. Adjust the filtrate to neutrality with acetic acid and vacuum-concentrate to a viscous consistency. Titrate the viscous liquid into fivefold the volume of ethanol under stirring. Allow to stand for at least 6 h before collecting the precipitated hemicellulose. Dialyse for at least 5 days (using dialysis bags with a molecular weight cut-off of 3000-8000). After dialysis, collect all liquid from the dialysis bag. Vacuum-concentrate and freeze-dry to obtain the hemicellulose sample.

S1.5 Structure elucidation of the substrates after OCF

The chemical compositions (%, w/w) of the substrates after OCF were determined according to the NREL standard analytical method (NREL/TP-510-42618). The 0.3 g power was subjected to 3 mL of 72% sulfuric acid hydrolysis at 30°C for 1 h under stirring, followed by diluting the acid to 4% concentration by adding 84 mL deionized water and further hydrolysis at 121°C for 1h. After the reaction, the hydrolysis solution was vacuum filtered through the previously weighed filtering crucibles (G3). The acid soluble lignin (ASL) in the filtrate solution was quantified by UV-Visible spectrophotometer (TU-1901, Persee) at 240 nm. The filtrate solution was diluted with deionized water to bring the absorbance into the range of 0.7-1.0, and the same concentration of sulfuric acid solution was used as the blank. The acid insoluble residue in filtering crucibles was rinsed with distilled water until the washing solution was neutral to remove the glucose from acid insoluble residue, which was determined to be acid insoluble lignin (AIL) and weighted after drying. The released monosaccharides in the filtrate solution were quantified by high performance anion exchange chromatography (HPAEC, Dionex ISC-3000, USA).

The cellulose retention, hemicelluloses retention and lignin recovery were calculated as follows:

$$\text{Lignin DCM oil (wt\%)} = \frac{\text{DCM extracted oil quality}}{\text{poplar lignin quality}}$$

$$\text{Solid recovery (wt\%)} = \frac{\text{OCF treated solid quality - catalyst quality}}{\text{poplar quality}}$$

$$\text{Delignification (wt\%)} = 1 - \frac{\text{lignin quality after OCF} \times \text{solid recovery}}{\text{poplar lignin quality}}$$

$$\text{Cellulose retention (wt\%)} = \frac{\text{cellulose quality after OCF}}{\text{poplar cellulose quality} \times \text{solid recovery}}$$

$$\text{Hemicellulose retention (wt\%)} = \frac{\text{hemicellulose quality after OCF}}{\text{poplar hemicellulose quality} \times \text{solid recovery}}$$

$$\text{Monomers yield (wt\%)} = \frac{\text{monomers quality}}{\text{poplar lignin quality}}$$

S1.6 Characterization of the lignin fractions

The molecular weight of lignin DCM oil and poplar DEL lignin were detected by gel permeation chromatography (GPC, Agilent 1200, USA) with an ultraviolet detector (UV) at 240 nm. Before the GPC detection, the lignin fraction was firstly acetylated in a DMSO/NMI dissolution system. Specifically, 20 mg of sample was dissolved in DMSO: N-Methylimidazole (2:1, v/v), the solution placed on a room temperature with a dark environment shaker 24 h. When sample dissolved completely, 0.4 mL acetic anhydride was added into the solution for an additional 1.5 h. At the end of the designated time, the solution was dropped slowly into 120 mL of acid water (pH = 2.0) adjusted by HCl to induce precipitation. The precipitate was washed and freeze-dried as acetylated lignin.

Lignin DCM oil was dissolved in methanol with 5 μL of n-decane as an internal standard and filtered through a 0.22 μm filter. The lignin DCM oil was analyzed by GC-MS (Agilent 8860 GC system equipped with an HP-5 MS column and Agilent 5977C Mass Spectrometer detector). The following GC or GC-MS procedure was used: 1 μL

aliquot with a split ratio of 50:1, injection temperature of 300 °C, column temperature program: 40 °C (hold 1 min), 10 °C min^{-1} to 300 °C (hold 5 min), detection temperature of 325 °C. The conversion and selectivity were calculated based on the numbers of the moles of product.

The detailed structural informations of lignin samples were detected by 2D-HSQC NMR technique on a Bruker AVIII 400 MHz spectrometer (Bruker, Germany). 20 mg of lignin was dissolved in 0.5 mL of DMSO-d_6 (99.8%), and the Bruker standard pulse program hsqcetgp was used for 2D-HSQC experiments. The spectral widths were 5000 and 20000 Hz for the ^1H and ^{13}C dimensions, respectively. The number of collected complex points was 1024 for ^1H-dimension with a recycle delay of 1.5 s. The number of transients was 64- and 256-time increments were always recorded in the ^{13}C dimension. Prior to Fourier transformation, the data matrixes were zero-filled to 1024 points in the ^{13}C dimension. All the NMR data processing were managed by standard Bruker Topspin-NMR software (Topspin 2.1).

S1.7 Characterization of the hemicellulose

The molecular weights of the hemicelluloses samples were determined by gel permeation chromatography (GPC, Agilent 1200, USA) on a PL aquagel-OH 50 column (300 × 7.7 mm, Polymer Laboratories Ltd.). The data were calibrated with PL pullulan polysaccharide standards (peak average molecular weights 180, 9600, 107,000, 708,000, Polymer Laboratories Ltd.). Detection was achieved with a Knauer

differential refractometer. The eluent was 0.02 M NaCl in 0.005 M sodium phosphate buffer (pH = 7.5). A flow rate of 0.5 mL/min was maintained. The column oven was kept at 30°C. Hemicelluloses samples were dissolved with 0.02 M NaCl in 0.005 M sodium phosphate buffer, pH = 7.5, at a concentration of 0.1%.

The detailed structural information of hemicelluloses macromolecules was detected by 2D-HSQC NMR technique on a Bruker AVIII 400 MHz spectrometer (Bruker, Germany). Before the test, 50 mg of hemicelluloses macromolecules was added to 0.5 mL D_2O, and 3 drops of deuterated sodium hydroxide (7.5 M, NaOD) were added to increase the solubility of hemicelluloses. The 2D-HSQC NMR spectra were acquired in the HSQCETGP experiment mode at 400 MHz. The spectral widths were 2200 Hz for the ^{1}H- (1024 data points) and 15,400 Hz for the ^{13}C dimensions with 256 times increments using 32 scans, with a recycle delay of 1.5 s. The $^{1}J_{C-H}$ used was 146 Hz. Prior to Fourier transformation, the data matrixes were zero filled up to 1024 points in the ^{13}C dimension. The solution-state ^{13}C NMR spectra were recorded at 25°C after 30,000 scans in the FT mode at 100.6 MHz, with a 30° pulse flipping angle and 9.2 μs pulse width. The acquisition time was 1.36 s, with a relaxation delay time of 2 s. All the NMR data processing were managed by standard Bruker Topspin-NMR software (Topspin 2.1).

S2. Tables and Figures

Table S1. Lignin DCM oil yield under OCF different conditions.

Temperature (°C)	Lignin DCM oil (wt%)	Time (h)	Lignin DCM oil (wt%)	Air pressure (MPa)	Lignin DCM oil (wt%)
160	44.59	3	61.54	1	52.06
180	57.36	4	72.19	1.5	72.19
200	72.19	5	62.51	2	63.23
220	53.23	8	54.23	2.5	58.67
240	46.84	12	44.57	2.5 (N_2)	41.37

Catalyst dosage (mg)	Lignin DCM oil (wt%)	Solvent	Lignin DCM oil (wt%)	Co molar ratio	Lignin DCM oil (wt%)
0	3.32	Methanol	72.19	5%	35.87
500	43.51	Ethanol	65.27	10%	44.78
200	63.33	EG	0	15%	60.61
100	72.19	Glycerol	0	20%	72.19
50	63.47	-	-	25%	50.19

Table S2. BET surface area (m^2/g), Pore diameter (nm) of Co-LDO with different Co doping ratios.

Sample	BET surface area (m^2/g)	Pore diameter (nm)
5% Co Molar Ratio	98.52±0.60	5-45
10% Co Molar Ratio	187.25±0.72	5-35
15% Co Molar Ratio	196.36±0.65	5-25
20% Co Molar Ratio	285.82±0.56	1-5
25% Co Molar Ratio	171.67±0.46	5-35

Table S3. Chemical composition (Cellulose, Hemicellulose, Lignin) of the different LDOs.

Sample	Cellulose	Hemicelluloses	Lignin	Others
Poplar	49.12±1.12	20.25±1.25	27.93±1.45	2.70±0.02
Con	62.38±0.75	19.66±0.91	14.84±1.01	3.12±0.03
MgAl-LDO	64.33±0.97	19.33±0.42	14.06±0.61	2.28±0.02
Cu-LDO	70.59±1.02	20.39±0.26	7.96±0.26	1.06±0.02
Ni-LDO	67.11±1.13	20.01±0.19	11.00±0.22	1.88±0.03
Fe-LDO	68.78±0.88	21.24±0.38	8.13±0.35	1.85±0.01
Co-LDO	72.34±0.69	22.51±0.21	2.51±0.29	2.64±0.03

Table S4. Solid yield, Delignification, Hemicellulose retention, Cellulose retention, Lignin DCM oil (wt%) of the different LDOs.

Sample	Solid yield	Delignification	Hemicellulose retention	Cellulose retention	Lignin DCM oil
Poplar	-	-	-	-	-
Con	77.73	58.70±0.35	75.47±0.79	98.71±0.84	3.32
MgAl-LDO	61.47	69.06±0.42	58.68±0.51	80.50±0.56	35.87
Cu-LDO	59.91	82.93±0.65	60.32±0.46	86.10±0.64	51.85
Ni-LDO	60.09	76.33±0.57	59.38±0.55	82.10±0.72	56.74
Fe-LDO	60.36	82.43±0.69	63.31±0.46	84.52±0.65	64.02
Co-LDO	66.70	94.01±0.76	74.14±0.42	98.23±0.62	72.19

Table S5. Assignments (ppm) of ^{13}C-^{1}H cross signals in 2D-HSQC NMR spectra of hemicellulose fractions.

Glycosyl	Assignments (ppm)							
		1	2	3	4	5eq [c]	5ax [d]	OCH_3
X [a]	^{13}C	102.9	73.0	74.4	76.0	63.1	63.0	NA [e]
	^{1}H	4.3	3.14	3.36	3.62	3.93	3.22	NA
U [b]	^{13}C	97.4	71.4	72.4	82.5	72.0 [f]	NA	59.6
	^{1}H	5.16	3.43	3.61	3.06	4.19	NA	3.34

[a] X, (1→4)-*β*-D-Xylp.

[b] U, 4-*O*-methyl-α-D-GlcpA.

[c] eq, equatorial.

[d] ax, axial.

[e] NA not assigned.

[f] C_5-H_5 in U has one cross peak because only one proton is linked to C-5

Table S6. Molecular weights of the hemicelluloses.

Sample	M_w (g/mol)	M_n (g/mol)	PDI
H-Poplar	107940±30	38460±20	2.81
H-Con	89640±50	32520±40	2.76
H-Co-LDO	86830±20	31570±10	2.75

Table S7. Assignments (ppm) of ^{13}C-^{1}H cross signals in 2D-HSQC NMR spectra of lignin fractions.

Label	δ_C/δ_H (ppm)	Assignments
C_γ	53.1/3.46	C_β-H_β in phenylcoumaran substructures (C)
B_β	53.5/3.05	C_β-H_β in β-β (resinol) substructures (B)
OCH_3	56.4/3.70	C-H in methoxyls (OMe)
A_γ	59.9/3.35-3.80	C_γ-H_γ in c-hydroxylated β-*O*-4′ substructures (A)
A'_γ	63.0/4.36	C_γ-H_γ in γ-acylated β-*O*-4′ substructures (A′)
C_γ	62.2/3.76	C_γ-H_γ in β-β′ resinol substructures (C)
I_γ	61.2/4.10	C_γ-H_γ in cinnamyl alcohol end-groups (I)
B_γ	71.0/3.79-4.16	C_γ-H_γ in β-β resinol substructures (B)
A_α	71.8/4.86	C_α-H_α in β-*O*-4′ substructures (A)
D_α	81.0/5.10	C_α-H_α in spirodienones (D)
C_α	86.8/5.45	C_α-H_α in phenylcoumaran (C)
B_α	84.8/4.66	C_α-H_α in β-β resinol (B)
A_β(G)	83.4/4.38	C_β-H_β in β-*O*-4 substructures linked to a G unit (A)
A_β(S)	85.8/4.12	C_β-H_β in β-*O*-4 linked to S (A, *erythro*)
A_β(S)	86.7/4.00	C_β-H_β in β-*O*-4 linked to S (A, *threo*)
$S_{2,6}$	103.9/6.70	$C_{2,6}$-$H_{2,6}$ in syringyl units (S)
$S'_{2,6}$	106.3/7.32	$C_{2,6}$-$H_{2,6}$ in oxidized S units (S′)
G_2	110.8/6.97	C_2-H_2 in guaiacyl units (G)
G_5	114.5/6.70	C_5-H_5 in guaiacyl units (G)
G_6	119.0/6.78	C_6-H_6 in guaiacyl units (G)
$PB_{2,6}$	131.3/7.65	$C_{2,6}$-$H_{2,6}$ in *p*-hydroxybenzoate (PB)
SM/GM_γ	72.8/4.01	C_γ-H_γ in SM/GM
SM/GM_β	123.4/6.17	C_β-H_β in SM/GM
SM/GM_α	132.6/6.48	C_α-H_α in SM/GM
G'_2	111.2-113.1/ 7.39-7.44	C_2-H_2 in guaiacyl units (G′)
G'_6	123.9-129.9/ 6.76-6.96	C_6-H_6 in guaiacyl units (G′)
